# Influence of Applied Liquid-Type Scanning-Aid Material on the Accuracy of the Scanned Image: An In Vitro Experiment

**DOI:** 10.3390/ma13092034

**Published:** 2020-04-27

**Authors:** Hyun-Su Oh, Young-Jun Lim, Bongju Kim, Won Hyeon Kim, Myung-Joo Kim, Ho-Beom Kwon

**Affiliations:** 1 Department of Prosthodontics and Dental Research Institute, School of Dentistry, Seoul National University, Seoul 03080, Korea; hsoh1391@snu.ac.kr (H.-S.O.); silk1@snu.ac.kr (M.-J.K.); proskwon@snu.ac.kr (H.-B.K.); 2 Dental Life Science Research Institute & Clinical Translational Research Center for Dental Science, Seoul National University Dental Hospital, Seoul 03080, Korea; wonhyun79@gmail.com

**Keywords:** intraoral scanners, trueness, precision, root mean square (RMS), scanning-aid materials

## Abstract

The study was designed to evaluate the effects of a liquid-type scanning-aid material on the accuracy and time efficiency of intraoral digital impressions compared to those of two different types of powder scanning-aid material and the powder-free scanning method. Three reference models (inlay, onlay, and bridge) were fabricated by a 3D printer and scanned with a model scanner to make the reference datasets. Four experimental groups (application of ScanCure, VITA, IP, and no treatment) were established, and the scans were acquired (each n = 5) using the Trios 3® (3 Shape, Copenhagen, Denmark). All scan data were digitally superimposed with the reference data (trueness, n = 5), and group comparisons were performed for each group (precision, n = 10). Time efficiency was evaluated by comparing the working times for scanning the models. The liquid-type ScanCure group showed fewer errors than the IP and VITA groups in all three reference models. Particularly, in the inlay model, the ScanCure group showed high accuracy compared to the powder-type groups (IP and VITA) with statistical significance (*p* < 0.001). The working time of the no-treatment group was longer than that of the agent groups in all reference models (*p* < 0.001). Notably, in the bridge model, the working time of the ScanCure group was shorter than that of the IP and VITA groups. Unlike other spray-type scanning-aid materials, this liquid-type material has the advantage of being thinly and uniformly applied to the object surface at the time of use. These findings suggest that the liquid-type scanning-aid material would be more accurate in achieving shape reproducibility using an intraoral scanner than the other two spray-type groups.

## 1. Introduction 

In daily clinical practice, digital impression with an intraoral scanner has been widely used for its advantages in allowing for reduced storage space, short laboratory time, no distortion errors from the impression material, patient comfort, better hygiene, and long-term low cost [1,2,3].

Most dental intraoral scanners currently available on the market are powder-free types, which means that they do not require any scanning spray. However, their clinical efficiency is for only a short span [4,5]. It is challenging to quickly obtain accurate data over a long span, such as a complete arch [6,7,8,9]. Furthermore, in some clinical situations, it is challenging to obtain reliable and precise data for the necessary parts in the narrow and deep areas of the prepared teeth and prostheses using metallic materials because of the reflection of light, even though it is for a short span [10].

The intraoral optical scanners used in the field of dentistry are extremely sensitive to glossiness and the transparency of the scanned surfaces [11,12,13]. A solution that can be considered to reduce these properties is to apply a scanning spray on the tooth or prosthesis because it enhances the opacity of the scanned surface and makes the dental surface the same color and opaque without any reflections. Therefore, some intraoral scanners recommend the use of a scanning spray for all digital impressions in real clinical situations.

However, it is hard to make a uniform layer of powder by spraying because the amount of applied powder is greatly affected by the operator’s skills, the presence of saliva and the tongue, and the space between the dental arches [14]. Consequently, the uneven accumulation of powder on the surface of the prepared teeth has adverse effects on the cement space and marginal fitness of the restoration, thus resulting in a poor clinical outcome [11]. Although the small size of the spray particles could probably allow for thin and uniform coating of the scan spray, the application of these sprays using a conventional pump injector may distort the geometry of the scanned surface [11]. Therefore, the powder needs to be applied “as thin as possible”, and the spraying time needs to be “as short as possible” [13].

The harmful effects of titanium dioxide on patients and dental staff, as their respiratory organs are exposed to fine or ultrafine particles, also pose a challenge. However, this can be effectively prevented by using a high-volume vacuum system [15].

Recently, the liquid-type scanning-aid material has been used because it is absorbed into the scanned surface and helps to make a thinner and a more uniform layer compared to the powder-type spray.

The objective of this study is to evaluate the influence of the liquid-type scanning-aid material on the accuracy (trueness and precision) of intraoral digital impressions. Additionally, the time efficiency of scanning for each of the three different types of scanning-aid materials was investigated in comparison to powder-free scanning.

## 2. Materials and Methods

### 2.1. Reference Models

**Figure 1:**
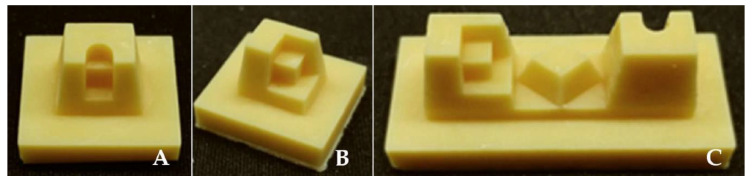
Reference models fabricated by 3D printer: (**A**) inlay model, (**B**) onlay model, (**C**) bridge model.

Reference models were designed using a Computer Aided Design (CAD) software (Solidworks 2016^TM^, Dassault Systèmes SolidWorks Corp., Waltham, MA, USA), and were fabricated with a 3D printer using an additive type photo-curing molding machine (Perfactory Micro 3D Printer®, EnvisionTec, Dearborn, MI, USA) (Figure 1). The material used for 3D printing was E-Denstone® (EnvisionTec, Dearborn, MI, USA).

These reference models were explicitly created to evaluate the accuracy of the digital impressions acquired using an intraoral scanner and were designed to reproduce the inlay, onlay, and crown shapes for dental restoration. The bridge model was implemented by connecting these three types.

One 3D printing model was produced for each reference model, and scanned with Identica Hybrid® (Medit Co, Seoul, Korea) model scanner to establish reference datasets. Then, using the same model, a total of 5 scan images were obtained for each group (application of Scan Cure® (ScanCure), IP Scan spray® (IP), Vita Powder Scan Spray® (VITA), and no treatment) using the TRIOS 3® (3 Shape, Copenhagen, Denmark) scanner to compare the reference datasets and models with scanning-aid materials to those with no scanning-aid materials. All scan data were digitally superimposed with the reference data (trueness, n = 5), and group comparisons were performed for each group (precision, n = 10). 

The cleaning step was performed by removing the scanning-aid material on the surface of the model by rubbing it with cotton balls soaked in organic solvent, removing the remaining residue with the air compressor water gun, and then drying the reference model. Then, the experiment was resumed.

### 2.2. Scanning-Aid Materials

In this study, three different types of scanning-aid materials were used: Scan Cure® (SC-80, ODS Co, Incheon, Korea), IP Scan spray® (IP-Division, Haimhausen, Germany) and Vita Powder Scan Spray® (Vita Zahnfabrik, Stuttgart, Germany). The product information is presented in Table 1.

### 2.3. Acquisition of Scanned Data and 3D Comparison

The reference models were digitally scanned with the TRIOS 3® (3 Shape, Copenhagen, Denmark) intraoral scanner. The scanned datasets of all experimental groups (ScanCure, IP, VITA, and no treatment) were converted to standard tessellation language (STL) file formats. These STL files were loaded into 3D analysis software (Geomagic Control X^TM^, 3d systems, Rock hill, SC, USA). Then, all datasets were superimposed with the reference dataset using the ‘best-fit method’ of the software. The alignment was set to produce a minimal error based on the least square regression with a set tolerance of ± 15 µm and a maximum tolerance range of ± 300 µm. The mean and standard deviation of the experimental groups were calculated using the root mean square (RMS) value, which is an indicator of the difference in displacement.

### 2.4. Comparison of Working Time

The time efficiency was evaluated by comparing the working time of the model scanning with no treatment and the application of the three types of scanning-aid materials.

### 2.5. Statistical Analysis

Statistical analysis was performed using the using the SigmaPlot 14.0^TM^ (Systat Software Inc., San Jose, CA, USA) program. Differences between groups in trueness, precision, and working times were evaluated by Kruskal–Wallis test. Pairwise comparisons were performed through the Mann-Whitney test in the case of significant difference according to the Kruskal–Wallis test. The level of significance (α = 0.05) was adjusted according to the Bonferroni correction method.

## 3. Results

### 3.1. Trueness

**Figure 2:**
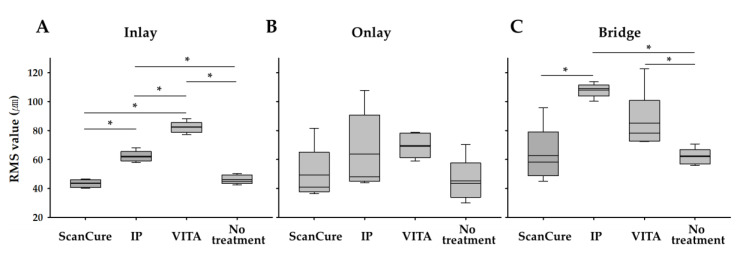
Box plots of the RMS values (trueness, n = 5) for four experimental groups (ScanCure, IP, VITA, no treatment, * *p <* 0.0083 by Bonferroni correction method) in three reference models. (**A**) inlay model, (**B**) onlay model, (**C**) bridge model.

The STL datasets were obtained using one intraoral scanner (Trios) and a model scanner (Identica) for the three reference model groups. In each of the three reference models, five sets of data (trueness, n = 5) were measured for each of the four experimental groups (ScanCure, IP, VITA, and no treatment), and the RMS values were analyzed. The trueness results are summarized in Table 2 and Figure 2. 

**Figure 3:**
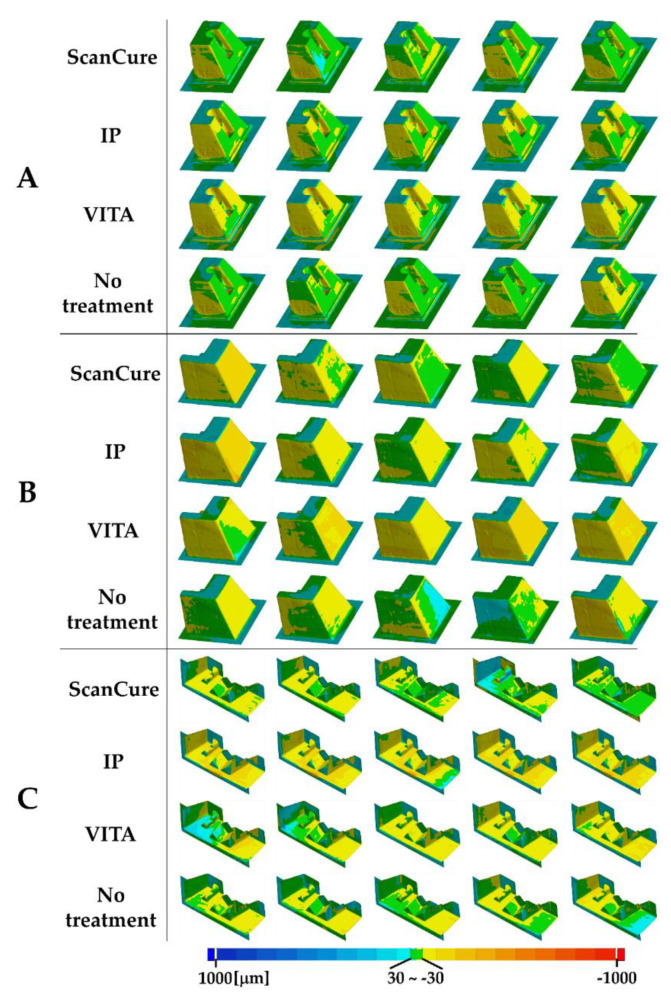
Superimposed error distribution for four experimental groups in the reference models. (**A**) inlay model, (**B**) onlay model, (**C**) bridge model (tolerance range ± 30 µm, respectively).

Overall, the ScanCure group showed fewer errors than the IP and VITA groups in all three reference models (Figure 2 and Figure 3). Particularly, in the onlay reference model, no statistically significant differences were seen among the three scanning-aid materials (ScanCure, IP, VITA). However, in the inlay reference model, the ScanCure group showed a statistically significant difference compared to the other two groups (IP and VITA) (*p* = 0.008). Furthermore, in the bridge reference model, the ScanCure group showed a statistically significant difference compared to the IP (*p* = 0.08).

In all three reference models, the trueness value in the no-treatment group was similar to the ScanCure group with no statistically significant differences. Therefore, it was believed that the ScanCure group would be more accurate in achieving shape reproducibility using an intraoral scanner than the other two groups (IP and VITA).

### 3.2. Precision

**Figure 4:**
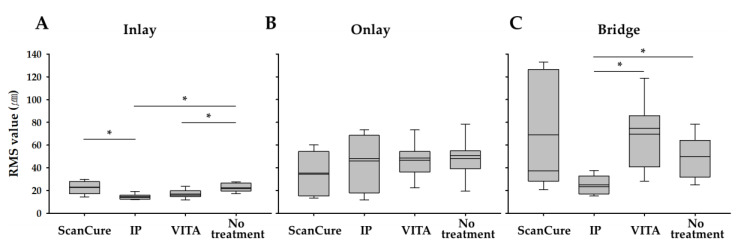
Box plots of the RMS values (precision, n = 10) for the four experimental groups (ScanCure, IP, VITA, no treatment, * *p <* 0.0083 by Bonferroni correction method) in three reference models. (**A**) inlay model, (**B**) onlay model, (**C**) bridge model.

STL datasets were obtained from the three reference model groups using one intraoral scanner (Trios). Ten RMS values (precision, n = 10) per group were analyzed with the combination of two different data for each group of five data, and the means and standard deviations are summarized in Table 3 and Figure 4.

In the inlay reference model, the IP and VITA groups showed a statistically significant lower RMS value than the no treatment group (*p* = 0.008, Figure 4A). Notably, in the onlay reference model, no statistically significant differences were found among the four groups (Figure 4B). Furthermore, in the bridge reference model, the IP group showed a statistically significant difference compared to the VITA and the no treatment groups (*p* = 0.001 and *p* = 0.003, respectively, Figure 4C). 

In the comparison of only three types of scanning-aid materials, the ScanCure showed a statistically higher precision RMS value compared to the IP and VITA groups in the inlay reference model (*p* = 0.008, Figure 2), while the IP group showed a statistically lower precision RMS value compared to the VITA group in the bridge reference model (*p* = 0.001, Figure 4).

### 3.3. Time Efficiency

**Figure 5:**
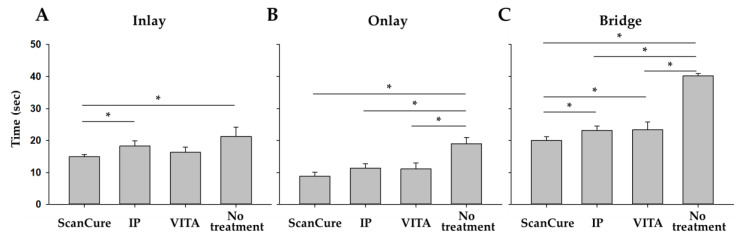
Bar graph of the working times for the four experimental groups (ScanCure, IP, VITA, No treatment, * *p* < 0.0083 by Bonferroni correction method) in three reference models. (**A**) inlay model, (**B**) onlay model, (**C**) bridge model.

The scanning times of the reference models in each experimental group were measured and summarized in Table 4 and Figure 5.

The working times of the no treatment group were longer than those when applying the agent groups in onlay and bridge reference models (*p* < 0.0083, Figure 5). Particularly, in the bridge model, the working time of the ScanCure group was shorter than that of the other agent groups (IP, VITA) (*p* < 0.0083, Table 4 and Figure 5). In addition, regardless of whether the agents were applied or not, the scan time increased in the order of onlay, inlay, and bridge with a statistical significance (*p* < 0.0167 by Bonferroni correction method, Figure 5).

## 4. Discussion

In previous studies, trueness and precision were measured to evaluate the accuracy of the intraoral scanner [16,17,18,19]. Trueness describes the deviation of the measured value from the original value, whereas precision describes the closeness between repeated measured values [16,17]. In this study, trueness and precision were assessed to compare the errors among three different scanning aid materials and no treatment.

### 4.1. Trueness and Precision

The results for trueness of the three different scanning agents show that the ScanCure group had higher trueness than the other groups. However, the results for precision showed that the IP group had higher precision than the other groups. The trueness and precision of the ScanCure group were similar to those of the no treatment groups, or the trueness and precision of the ScanCure group were higher in all three reference models (inlay, onlay, and bridge) with no statistically significant differences. Notably, in the inlay and bridge models, the ScanCure group had statistically lower errors than the other groups. Based on these results, it can be said that ScanCure is suitable for reproducing narrow and deep areas of the models.

**Figure 6:**
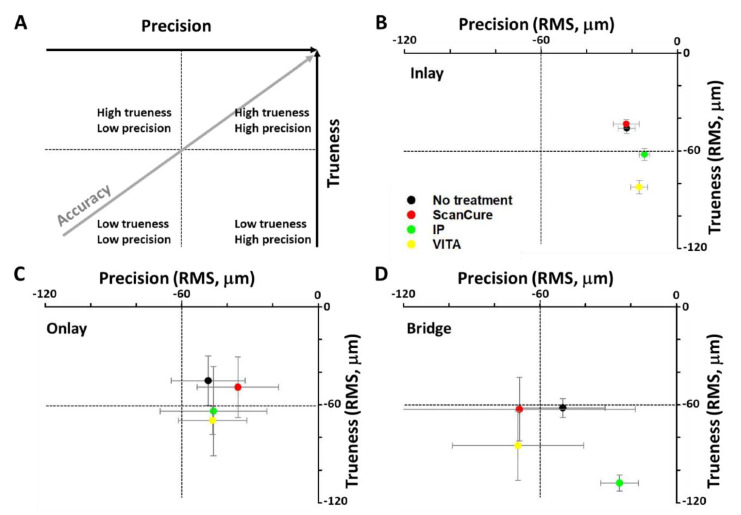
Accuracy of four experimental groups (ScanCure, IP, VITA, no treatment) in the inlay, onlay and bridge reference models. (**A**) Scheme of the correlation between precision and trueness, (**B**) inlay model, (**C**) onlay model, and (**D**) bridge model.

Figure 6 represents the analysis of the accuracy based on the trueness and precision results, and it also shows that the accuracy had a linear relationship with trueness and precision. In the inlay and onlay models, the Sca Cure and no treatment groups had higher trueness and precision. In contrast, the IP and VITA groups had lower trueness and precision. Therefore, higher accuracy could be obtained when ScanCure is applied in an actual clinical situation. 

Notably, in the bridge model, all experimental groups had low trueness with no statistically significant difference; however, the IP group had high precision. The IP group in the bridge model showed the largest error (more than 40 µm) compared to the other groups, and the error (less than 40 µm) was distributed across the flat area of the lowest part of the model (Figure 3C). Therefore, the IP group had lower trueness but higher precision than the other groups because it generally had high but similar errors in all three reference models (Figure 6D).

Uhm et al. [20], stated that the accuracy of the inlay and four-unit bridge models were analyzed by four different intraoral scanners and that the errors in the inlay model were more than two times those in the bridge model. Similarly, this study shows that the bridge model had higher errors of trueness and precision, compared to the single models like the inlay and onlay. This is because the bridge model had a larger area that needed to be scanned, unlike the inlay and onlay models. Furthermore, it was believed that high errors of the trueness and precision in the bridge model were caused by unnecessary areas outside the area of interest scanned by the intraoral scanner.

### 4.2. Comparison in Visual Between Scanning-Aid Materials 

The ideal requirements for scanning-aid materials include enhanced opacity of the model surface without a specific volume change and the acquisition of accurate and fast scan data.

Intraoral scanners recognize the shape of objects by detecting the light reflected from the surface of the objects [21]. Therefore, the scanning quality can be affected by reflective properties, such as the glossiness and transparency of the surface. The glossy and translucent features of the dentition make it challenging for the intraoral scanner to recognize the surface; thus, it produces a less accurate impression [11,13].

Moreover, it is challenging to scan the surface of a polished metal prosthesis because it has a highly reflective appearance [11]. To overcome these challenges, titanium dioxide powder can be used to enhance the opacity of the surface and make a uniform reflection of the light [13,22]. However, if the powder is over-applied on the tooth surface and the layer is thick, the scanner is not able to record the surface accurately [23]. This can result in undesirable gaps between the tooth preparation margin and the prosthesis [11].

The amount of powder applied is dependent upon the controls (amount, duration, and distance) of the operator [13]. In addition, saliva may immediately wash the powder away. These issues make it difficult to maintain the thin layer of powder uniformly over the tooth [24]. In addition, the powder is often unpleasant to both patients and operators.

**Figure 7:**
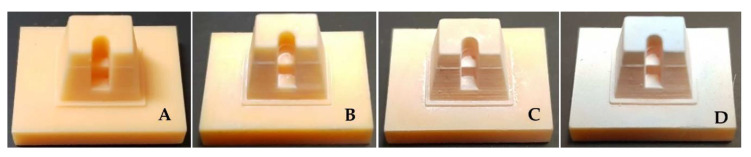
The inlay reference models with no treatment and 3 different scanning aid materials. (**A**) no treatment; (**B**) ScanCure; (**C**) IP; (**D**) VITA.

It was confirmed that the models fabricated with painting or spraying type scanning-aid materials were more clearly distinguishable than the non-treated ones. (Figure 7). A layer applied on the model surface was checked with the naked eye and photos were taken to verify the difference between the spraying and painting types. It was found that when the liquid-type ScanCure was painted on the reference model, it penetrated the model surface and created a thin layer. However, it was visually verified that after spraying the powder-type IP and VITA, the particles accumulated on the model surface and created a thick layer compared to the layer created by the ScanCure (Figure 7). Therefore, it was considered that the liquid-type ScanCure had superior shape reproducibility compared to the powder-type materials (IP and VITA). 

### 4.3. Comparison in Working Time Between Scanning-Aid Materials

The scanning times with the application of the agent groups were shorter than that of the no-treatment group with a statistical significance because the intraoral scanner could recognize the uniform reflection of the model surface more efficiently when the agents were applied. In the bridge model, the liquid-type ScanCure group was more time-efficient than the other powder-type groups (IP and VITA).

These results might be explained by the liquid-type agents making the surface more uniform than the powder-type agents. In addition, the working time taken for the inlay model was longer than that of the onlay model because it was harder to recognize the internal structure of the inlay model covered by the four walls. In this study, even though one skilled prosthodontist measured the working time, the time for obtaining the digital impression was affected by the operator’s skill and experience. Moreover, there is a learning curve for operating 3D intraoral scanners [25]. In addition, in this experiment there were no considerations of potential errors imposed by patients, such as limited mouth opening, saliva, and movements of the tongue and cheeks. However, in real clinical situations, the operator and the patient’s factors affect the scanning time.

A limitation of this experiment was that it was not possible to simulate the phenomenon that usually occurs during the scanning of metallic materials with intraoral scans using resin-like materials in reference model making. Dental oral scanners are extremely sensitive to the optical properties of the scanned surfaces, particularly, the glossiness and transparency. Therefore, it is difficult and time-consuming to acquire data on metal and related parts. Thus, subsequent experiments using a metal reference model for confirmation are necessary.

## 5. Conclusions

The liquid-type scanning-aid material here tested has superior shape reproducibility than the powder-type scanning-aid material. Unlike other scanning-aid materials, this liquid-type material can be uniformly and thinly applied to the object surface with the brush application method at the time of use. Therefore, the liquid-type scanning-aid material can be strategically used to obtain fast and accurate data in situations or areas where it is difficult to obtain a scan image. 

## Figures and Tables

**Table 1:** Information on the scanning-aid materials

Commercial name	ScanCure®	IP Scan Spray®	Vita Powder Scan Spray®
Product	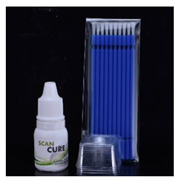	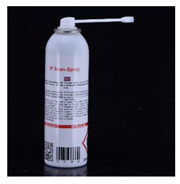	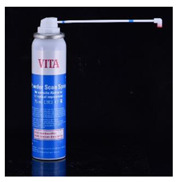
Method of application	painting	spraying	spraying
Type	liquid	powder	powder
Composition	Ethanol TiO_2_	Butane, Propane Ethanol Isobutane TiO_2_	Ethanol, Isobutane TiO2-free
Color	white	white	Light blue

**Table 2:** Mean trueness value according to an intraoral scanner (mean ± SD of RMS)

Trueness	n	ScanCure (µm)	IP (µm)	VITA (µm)	No Treatment (µm)
Inlay	5	43.44 ± 2.75	62.16 ± 3.77	82.28 ± 4.06	46.12 ± 3.23
Onlay	5	49.26 ± 18.63	63.90 ± 27.38	69.62 ± 8.60	45.26 ± 15.20
Bridge	5	62.78 ± 19.53	107.90 ± 4.87	85.08 ± 21.18	61.90 ± 5.74

SD, standard deviation. RMS, Root Mean Square.

**Table 3:** Mean precision value according to an intraoral scanner (mean ± SD of RMS)

Precision	n	ScanCure (µm)	IP (µm)	VITA (µm)	No Treatment (µm)
Inlay	10	22.60 ± 5.65	14.53 ± 2.36	16.83 ± 3.68	22.36 ± 3.66
Onlay	10	35.31 ± 17.90	46.04 ± 23.52	46.50 ± 15.14	48.39 ± 16.25
Bridge	10	69.08 ± 51.10	25.00 ± 8.28	69.67 ± 28.91	49.97 ± 18.54

SD, standard deviation. RMS, Root Mean Square

**Table 4:** Working times, according to scanning-aid materials (mean ± SD)

Time	n	ScanCure (sec)	IP (sec)	VITA (sec)	No Treatment (sec)
Inlay	5	14.92 ± 0.70	18.22 ± 1.68	16.30 ± 1.62	21.28 ± 2.90
Onlay	5	8.87 ± 1.23	11.32 ± 1.44	11.11 ± 1.87	18.97 ± 1.91
Bridge	5	20.01 ± 1.19	23.17 ± 1.34	23.37 ± 2.40	40.18 ± 0.69

SD, standard deviation.
